# COVID-19: A Boon or a Bane for Creativity?

**DOI:** 10.3389/fpsyg.2020.601150

**Published:** 2021-01-18

**Authors:** Maxence Mercier, Florent Vinchon, Nicolas Pichot, Eric Bonetto, Nathalie Bonnardel, Fabien Girandola, Todd Lubart

**Affiliations:** ^1^Université de Paris and Univ Gustave Eiffel, LaPEA, Boulogne-Billancourt, France; ^2^Aix-Marseille University, Center for Research on the Psychology of Cognition, Language and Emotion, Marseille, France; ^3^Institute of Creativity and Innovation of Aix-Marseille, Marseille, France; ^4^Laboratoire de Psychologie Sociale, Aix-Marseille University, Marseille, France

**Keywords:** lockdown, COVID-19, creativity, creativity development, positive outcome, little-c, Pro-C

## Abstract

In many countries, the COVID-19 pandemic led to a period of lockdown that impacted individuals’ lifestyles, in both professional and personal spheres. New problems and challenges arose, as well as opportunities. Numerous studies have examined the negative effects of lockdown measures, but few have attempted to shine light on the potential positive effects that may come out of these measures. We focused on one particular positive outcome that might have emerged from lockdown: creativity. To this end, this paper compared self-reported professional creativity (Pro-C) and everyday creativity (little-c) before and during lockdown, using a questionnaire-based study conducted on a French sample (*N* = 1266). We expected participants to be more creative during than prior to lockdown, in both professional and everyday spheres. Regarding Pro-C, we did not see any significant differences between the two comparison points, before and during lockdown. Regarding everyday creativity, we observed a significant increase during lockdown. Furthermore, our results suggest that participants with a lower baseline creativity (before lockdown) benefited more from the situation than those with a higher initial baseline creativity. Our results provide new insights on the impact of lockdown and its positive outcomes. These measures may have inarguably negative consequences on the physical and mental health of many, but their positive impact exists as well.

## Introduction

In the wake of the outbreak of the COVID-19 pandemic, many different governments imposed periods of lockdown, to restrict the movement of the population and thus reduce pressure on healthcare systems. These measures have been mostly effective in that regard (Alfano and Ercolano, 2020). However, they also took a toll on individuals’ physical and mental health (Brooks et al., 2020; World Health Organization, 2020), and impacted their lifestyles, in both personal and professional spheres. As such, the COVID-19 and ensuing lockdowns are having an unprecedented impact on social lives and are viewed by many as global stressors. Nonetheless, for all the numerous negative consequences of lockdown measures that have been studied so far, few efforts have attempted to shed light on their possible positive outcomes. Two defining aspects of lockdown situations are uncertainty (Nitschke et al., 2020) and solitude (Banerjee and Rai, 2020). One outcome that is frequently associated with these two elements is creativity (e.g., Long and Averill, 2003; Ford et al., 2008). In this study, we argue that, in spite of the negative outcomes that came out of it, lockdown may have fostered creativity in the general population, in France.

Creativity can be defined as the capacity to generate productions that are both novel and relevant (Runco and Jaeger, 2012). Novelty can be understood as the originality and unusualness of the production. Relevance can be understood in terms of usefulness (Runco, 1988), value (Ford, 1996), appropriateness (Runco et al., 2005), or ability to solve a specific problem (Besemer and Treffinger, 1981). Creativity varies in terms of scale, or magnitude: whereas a child’s poem composed for his/her mother and Walt Whitman’s *Song of Myself* are arguably both creative achievements in their own right, a certain degree of magnitude separates the two. The different graduations of creativity are conceptualized in the Four C model of creativity (Kaufman and Beghetto, 2009). This framework distinguishes four ordered classes of creativity, in descending order: Big-C, Pro-C, little-c, mini-c. Big-C refers to clear-cut, eminent creative accomplishments: examples might include Picasso’s *Guernica* and Marie Curie’s work with radium. They are eminent works that define or change a creative domain. Pro-C refers to professional-level accomplishments, that are performed by a domain’s practitioner, significant enough to contribute to this domain’s growth but not eminent within it. For instance, a jazz pianist may be able to make a living thanks to jazz classics and improvisations, but does not reach eminence like Bill Evans or Oscar Peterson. Professional creativity (Pro-C) can also take the form of an engineer finding new and cost-effective solutions to a problem (Cropley, 2015), or a flight attendant finding a creative way to deliver security instructions, to set a relaxed environment for passengers while gaining their attention (Waples and Friedrich, 2011). Little-c is often referred to as “*everyday creativity*.” This form of creativity is practiced by virtually everyone and can take place in all spheres of life. It can take the form of finding a new way to decorate one’s room or mixing cuisines to create a new dish for a meal with friends. Finally, mini-c refers to the novel and personally meaningful interpretations of experiences, actions, and events (Beghetto and Kaufman, 2007). For instance, making sense of a life situation for oneself, or generating a new poetic composition just for oneself, to express one’s own emotions. These mini-c manifestations might not be public or tangible, yet they are meaningful to the individual creator and reflect the creation of new ideas and knowledge (Cotter et al., 2018). We will focus our attention on two aspects of creativity, Pro-C and everyday creativity (little-c).

In France, the first strict lockdown lasted for 55 days (17 March 2020 to 11 May 2020). This led to heightened uncertainty in the population (Fletcher and Griffiths, 2020; Nitschke et al., 2020): shops and restaurants closing without a precise perspective of reopening, numerous individuals being temporarily laid-off, unsure safety of close relations, and general uncertainty regarding the duration of strict lockdown, which was extended twice in France. Uncertainty often leads to stress (Peters et al., 2017) and anxiety (Counsell et al., 2017; Chen et al., 2018), as observed throughout the COVID-19 outbreak. However, times of uncertainty are also catalysts for creativity (Beghetto, 2019). Indeed, although creativity is not needed at all times and in all places (Kaufman and Beghetto, 2013), it is most certainly welcome in such unprecedented circumstances. In that sense, creative action can be understood as a way to make sense of and cope with uncertainty (Ford, 1996; Beghetto, 2019). This usually entails challenging one’s old assumptions and trying new things. Uncertainty should therefore offer an opportunity for creativity to emerge. In organizational and entrepreneurial contexts, uncertainty and its concurrent management is at the root of creative and innovative endeavors (Ford et al., 2008; Blauth et al., 2014). Indeed, the entrepreneurial attitude of embracing unexpected events and uncertainty in new product development is key to creativity and innovation (Blauth et al., 2014). The creation of ventures can thus be seen as a process whereby an entrepreneur addresses uncertainty with action. Uncertainty motivates exploration and consideration of creative actions (Ford et al., 2008). This should also apply to personal spheres, whereby the disruption of routines and lifestyles should lead to opportunities to consider and implement creative actions.

A second defining aspect of lockdown is the ensuing solitude (Banerjee and Rai, 2020): individuals were unable to leave their home, could not physically rejoin their family or their close relations, and most employees resorted to telework instead of going to their usual worksite. Solitude is a distinct yet related concept from loneliness. Whereas solitude refers to an objective state of being alone, loneliness is a negative emotion that stems from a misfit between desired and achieved levels of social contact (Perlman and Peplau, 1981). Solitude appears to cause adverse effects on one’s physical and mental health, leading to increased anxiety and depression, and poor health behaviors (for a review, see Holt-Lunstad et al., 2015), most of which have been observed during lockdown. However, solitude is also frequently associated with creativity. Notably, there is a long-standing assumption that solitude fosters creativity (Simonton, 2000), which can be attributed, in part, to the numerous creative works that have emerged from periods of solitude, from Thoreau’s *Walden* to Electric Light Orchestra’s *Mr. Blue Sky*. Solitude should be beneficial to creativity, as it can notably enable freedom of spirit (Arieti, 1976). Long and Averill (2003) suggested two ways solitude could facilitate creativity. One way would be by stimulating “*imaginative involvement in multiple realities*” (i.e., by enabling imagination, daydream, and wonder). This is notably corroborated by the effect solitude has on imaginative involvement in Antarctic research teams (Barabasz, 1991). Another way solitude might foster creativity is through the adoption of alternative selves and self-transformation. Solitude should notably facilitate self-reflection and contemplation (Koch, 1994), which are key to the adoption of new behaviors (Long and Averill, 2003). Similar to uncertainty, this should favor creativity in both personal and professional spheres. Furthermore, solitude has been associated with boredom (e.g., Farmer and Sundberg, 1986; Spores, 1991), an experience that has been seen throughout state-imposed lockdowns in Europe and the United States (Brodeur et al., 2020). This is notably caused by the deprivation of activities and interpersonal interactions that characterize situations of solitude and social isolation. Like solitude, boredom has been associated with a range of negative outcomes (for a review, see Vodanovich, 2003). However, recent research has also demonstrated that boredom could be beneficial to creativity (e.g., Mann and Cadman, 2014), as it is “an alerting phenomenon that all is not well and something must be done” (Gaylin, 1979, p. 129). Creativity could thus be viewed as a way to cope with boredom, for example, to explore new ways to conduct a boring task, in an attempt to make it more engaging or interesting (Toohey, 2011). Concurrently, we expect the boredom accompanying lockdown measures to be beneficial for creativity.

The aim of the present study was to explore whether lockdown in France led to an increase of professional (Pro-C) and everyday creativity (little-C) compared to the prior baseline situation, while controlling for multiple variables linked to creativity. Our hypotheses were as follows. Professional (H1a) and everyday (H1b) creativity should be higher during lockdown than prior to it. Recent research has indicated that creativity training benefits less those individuals who have high baseline creativity (Meinel et al., 2019). Thus, there seems to be a certain learning curve for creativity, and participants with lower creativity may benefit more from the lockdown conditions than those who have a high baseline level of creativity. Accordingly, we hypothesized that differences of Pro-C (H2a), between pre-lockdown and during-lockdown Pro-C, should be higher for individuals who displayed less Pro-C prior to lockdown. We hypothesized also that differences of everyday creativity (H2b), between pre-lockdown and during-lockdown everyday creativity, should be higher for individuals who displayed less everyday creativity prior to lockdown. Differences between pre-lockdown and during-lockdown professional (H3a) and everyday (H3b) creativity should be positively linked to the perceived state of boredom (during lockdown). Additionally, telework has been on the rise in France during lockdown (Pullano et al., 2020). Telework has been linked to better creative performance compared to usual workplace situations (Vega et al., 2015). This effect could be explained by the fact that telework entails more control over one’s schedule (Hill et al., 1996) and allows for a more autonomous environment (Alge et al., 2006), as opposed to the greater monitoring in the usual workplace, both of which have been linked to increased Pro-C (respectively, Amabile et al., 2002; Alge et al., 2006). Thus, we posited the following: (H4) teleworkers should display higher gains in Pro-C than employees who worked at their usual worksite during lockdown.

## Materials and Methods

### Sample and Data Collection

We collected data from 1266 participants, who were recruited online through French social networks (*M*_*age*_ = 39.22, *SD*_*age*_ = 11.76, 9.5% male). All participants lived in France during lockdown, and 97.3% were French. Concerning the participants work situation, 51.8% were working during lockdown (*N* = 656), among whom 58.7% worked through telework, 25% worked in their usual worksite, and 16.3% were working through other means (such as satellite office or mobile work). The data collection respected the General Data Protection Regulation (EU) 2016/679 (GDPR). All participants were recruited during France’s strict lockdown period, from 28 April 2020 to 5 May 2020.

### Material

#### Professional Creativity During Lockdown

We assessed Pro-C with a 13-item scale developed by Zhou and George (2001), using a 100-point Visual Analog Scale (VAS) ranging from “not at all” to “absolutely.” The scale was initially designed for supervisors to assess employee creativity. The instructions were adapted into a self-report version, as they have demonstrated satisfactory validity (see Ng and Feldman, 2012). It was translated to French using a back-translation procedure^1^ (Brislin, 1986). A sample item is “*I am a good source of creative ideas*.” We used confirmatory factor analysis (CFA) to verify the unidimensionality of the scale. A single-factor model yielded good fit indices: *χ*^2^(65) = 392.75, *p* < 0.001, CFI = 0.957, TLI = 0.948, RMSEA = 0.088, SRMR = 0.025. The internal consistency was high (*α* = 0.96).

#### Professional Creativity Before Lockdown

We assessed Pro-C before lockdown using an adaptation of the previous workplace creativity scale (Zhou and George, 2001), in the past tense, with a 100-point VAS. We instructed participants to think about the 2 months prior to lockdown (January and February 2020). Using CFA, a single-factor model yielded good fit indices: *χ*^2^(65) = 361.86, *p* < 0.001, CFI = 0.962, TLI = 0.955, RMSEA = 0.083, SRMR = 0.025. Internal consistency was high (*α* = 0.96).

#### Everyday Creativity During Lockdown

We measured everyday creativity using an adaptation of Zhou and George’s (2001) scale, with a 100-point VAS. The items were modified to correspond to everyday situations. One item was deleted during this process, as it could not be adapted satisfactorily: “*I develop adequate plans and schedules for the implementation of new ideas*.” A sample item is “*I come up with new and practical ideas*.” French and English translations are available in the Supplementary Appendix A. Using CFA, a single-factor model yielded good fit indices: *χ*^2^(54) = 682.06, *p* < 0.001, CFI = 0.948, TLI = 0.936, RMSEA = 0.096, SRMR = 0.031. The resulting 12-item scale showed high internal consistency (*α* = 0.95).

#### Everyday Creativity Before Lockdown

We assessed everyday creativity before lockdown using a past tense adaptation of the previous 12-item scale, with a 100-point VAS. We instructed participants to think about the two months prior to lockdown (January and February 2020). Using CFA, a single-factor model yielded good fit indices: *χ*^2^(54) = 491.56, *p* < 0.001, CFI = 0.963, TLI = 0.955, RMSEA = 0.080, SRMR = 0.026. Internal consistency was high (*α* = 0.95).

#### Boredom During Lockdown

State boredom was assessed using the short form of the Multidimensional State Boredom Scale (MSBS-8; Hunter et al., 2016), with a 100-point VAS. This eight-item scale, measuring state boredom as a unidimensional construct, was translated through a back-translation procedure. A sample item is “*I feel like I’m sitting around waiting for something to happen*.” Using CFA, a single-factor model yielded acceptable fit indices: *χ*^2^(20) = 408.68, *p* < 0.001, CFI = 0.906, TLI = 0.869, RMSEA = 0.124, SRMR = 0.057. Internal consistency was good (*α* = 0.86).

#### Control Variables

We included four control variables: leisure time, perceived difficulty of lockdown, personality factors, and creative self-concept. Leisure time and perceived difficulty of lockdown were both assessed with single items (respectively, “Do you have leisure time?” and “How difficult does the lockdown feel to you?”), using a 100-point VAS (respectively, from “Not at all” to “Absolutely,” and from “Not difficult at all” to “Very difficult”). Big Five personality factors were assessed using the French version of the Ten-Item Personality Inventory (TIPI; Storme et al., 2016), with a 100-point VAS. This scale measures Openness, Conscientiousness, Extraversion, Agreeableness, and Emotional Stability, with two items per factor. It has shown acceptable temporal stability, and satisfactory convergent and divergent construct validity. Internal consistency, calculated through inter-item correlations, was comparable to Storme et al.’s (2016) results (for details, see Supplementary Appendix B). Creative self-concept is defined as the convictions about one’s creative abilities and the nature of creativity (Karwowski and Barbot, 2016). It can be understood as a multi-faceted construct, covering multiple characteristics such as creative metacognition, creative self-efficacy (CSE), and creative personal identity (CPI). Out of these numerous components, we focused on two that are linked to fluctuations of creativity: CSE and CPI (Tierney and Farmer, 2011; Karwowski, 2016). CSE and CPI were measured with the Short Scale of Creative Self (SSCS; Karwowski, 2012; Karwowski et al., 2018), using a 100-point VAS. The SSCS is an 11-item scale, with six items measuring CSE, and five items measuring CPI. Sample items are “*I am sure I can deal with problems requiring creative thinking*” and “*My creativity is important for who I am*,” respectively. It was translated using a back-translation procedure. Using CFA, a two-factor model yielded satisfactory fit indices: *χ*^2^(43) = 838.72, *p* < 0.001, CFI = 0.933, TLI = 0.914, RMSEA = 0.121, SRMR = 0.058. Both scales showed good internal consistency (αCSE = 0.86; αCPI = 0.95).

## Results

### Preliminary Analyses

#### Common Source Bias

Because we collected all data from a single source and used self-report measures, we checked for the presence of common source bias, using Harman’s single-factor test (Harman, 1976; Podsakoff and Organ, 1986). First, we conducted an exploratory factor analysis (EFA), using maximum likelihood extraction, on all items of our scales, to check whether a single factor would account for the majority of variance. Without rotation, the first factor explained 35.9% of total variance, below the recommended 40%. Then, we conducted a CFA on the same dataset, in which all items loaded on a single factor. The resulting solution yielded mediocre fit indices: *χ*^2^(3002) = 33,183.46, *p* < 0.001, CFI = 0.530, TLI = 0.518, RMSEA = 0.089, SRMR = 0.100. These analyses led to reduced concerns over the presence of common source bias.

#### Creativity Latent Profiles

**FIGURE 1:**
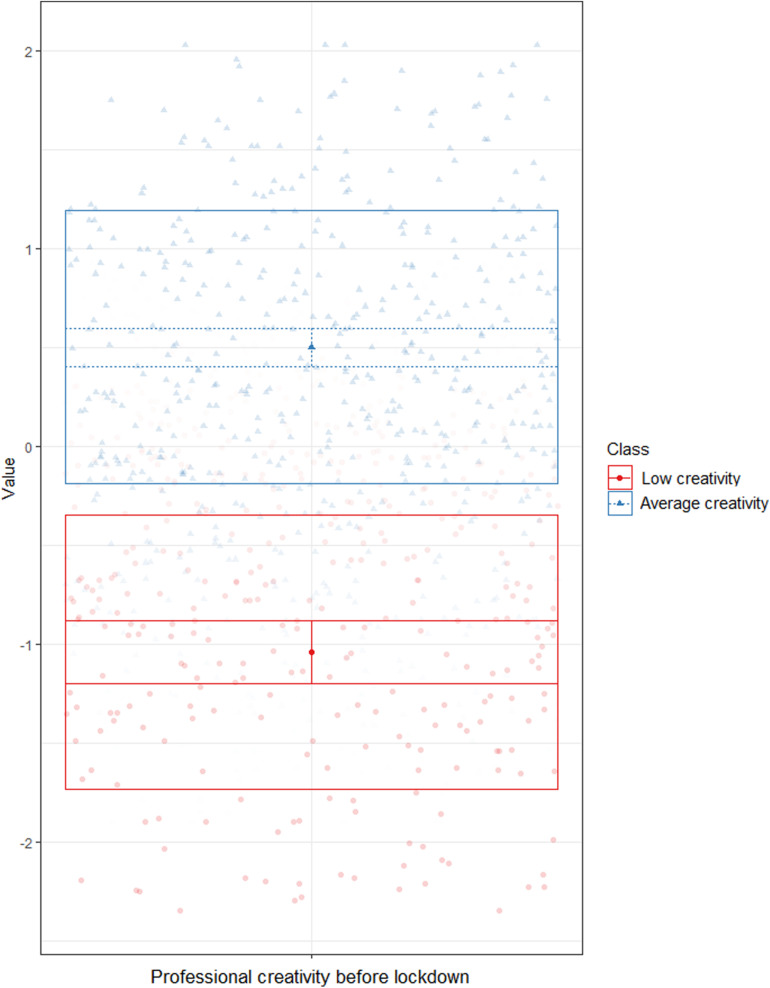
Latent profile analysis – Professional creativity before lockdown.

**FIGURE 2:**
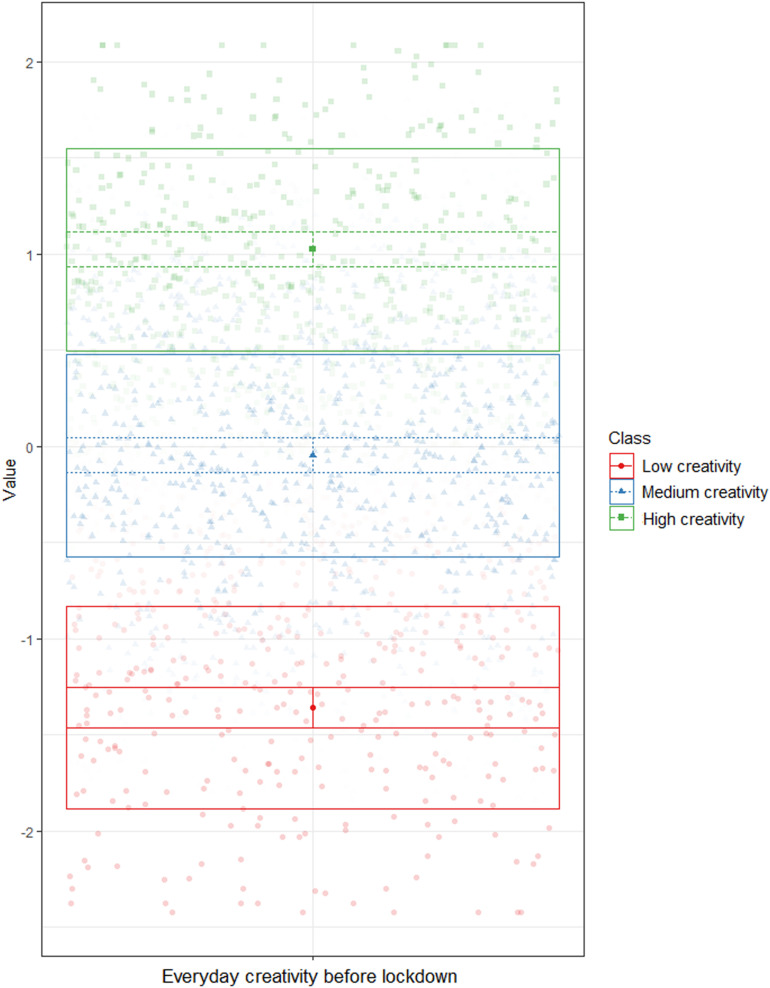
Latent profile analysis – Everyday creativity before lockdown.

We used latent profile analysis (LPA, Gibson, 1959) to estimate the creativity levels of our participants, based on self-reports of their professional and everyday creativity before lockdown. LPA is a clustering technique that identifies, in a given sample, groups (i.e., profiles) of individuals with similar values on variables of interest (Muthén, 2001). Similar to CFA, the optimal number of profiles is determined based on the analysis of fit indices. LPA compares iteratively an increasing number of profiles, to determine the optimal number. This optimal solution was determined using the analytical hierarchy procedure (AHP, Akogul and Erisoglu, 2017), which combines the analyses of five commonly used indices: Akaike’s information criterion (AIC, Akaike, 1998), approximate weight of evidence (AWE, Banfield and Raftery, 1993), Bayesian information criterion (BIC, Schwarz, 1978), classification likelihood criterion (CLC, Biernacki and Govaert, 1997), and Kullback’s information criterion (KIC, Cavanaugh, 1999). Akogul and Erisoglu (2017) demonstrated that AHP was more accurate in determining the optimal number of profiles, compared to the separate use of these criteria. After having determined the number of profiles, the corresponding profile is attributed to each individual. All analyses were conducted using R, with the tidyLPA package version 1.0.6 (Rosenberg et al., 2019). All scores were standardized prior to analyses. For Pro-C, we compared the results of seven solutions, from one to seven profiles (see Supplementary Table C1). Based on AHP, the optimal solution was the two-profile model (Figure 1). The two profiles were: low creativity (*N* = 205) and average creativity (*N* = 451). For everyday creativity, we compared the results of seven solutions, from one to seven profiles (see Supplementary Table C2). Based on AHP, the optimal solution was the three-profile model (Figure 2). The three profiles were: low creativity (*N* = 272), medium creativity (*N* = 626), and high creativity (*N* = 368).

### Professional Creativity

In order to test H1a, we calculated the difference between estimated Pro-C during and prior to lockdown and then conducted a matched sample (repeated measures) *t*-test. The *t*-test was not significant, *t*(655) = −1.26, *p* = 0.21, *M* = −0.92. Hypothesis 1a was not supported. There was no difference between creativity in the workplace before and during lockdown. To test H2a, we conducted a repeated measures ANOVA between creativity before lockdown and during lockdown, using the profile of creativity in the workplace as a factor. Consistent with the previous result, the ANOVA revealed no main effect of creativity circumstances on creativity, *F*(1,653) = 0.98, *p* = 0.32. There was no interaction between creativity circumstances and profile, *F*(1,653) = 0.16, *p* = 0.69. Thus, H2a was not supported. There was no significant correlation between creativity differences and state boredom (*r* = −0.03, *p* = 0.39). Thus, H3a was not confirmed. To test H4, we conducted a second repeated measures ANOVA, this time using work conditions during lockdown (usual worksite vs. telework) as a factor. The ANOVA revealed no main effect of creativity circumstances on creativity, *F*(1,516) = 3.67, *p* = 0.06. There was a significant interaction between time and work conditions, *F*(1,516) = 7.39, *p* < 0.01, *η*^2^ = 0.002, *η*^2^*_*p*_* = 0.01. *Post hoc* comparisons revealed no significant differences of creativity for teleworkers, *t*(516) = 0.72, *p*_*tukey*_ = 0.89. However, there was a significant increase of creativity for participants who had to work at their usual worksite, *t*(516) = 2.79, *p*_*tukey*_ < 0.05, *d*_*RM*_ = 0.2, *M*_*diff*_ = 3.98, *SE* = 1.43. Thus, H4 was not corroborated. In fact, the opposite tendency was observed.

### Everyday Creativity

**FIGURE 3:**
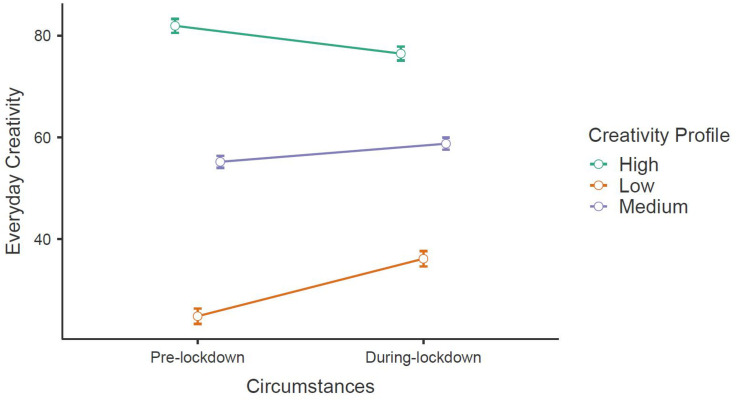
Change in everyday creativity, for low, medium, and high creativity profiles.

In order to test H1b, we calculated the difference between estimated everyday creativity during and before lockdown and then conducted a repeated measures matched-sample *t*-test. The *t*-test was significant, *t*(1265) = 5.4, *p* < 0.001, *d* = 0.15, *M*_*diff*_ = 2.6. These results were consistent with H1b. To test H2b, we conducted a repeated measures ANOVA between creativity before lockdown and during lockdown, using the profile of everyday creativity as a factor. Consistent with H2b, the ANOVA revealed a main effect of creativity circumstances on creativity, *F*(1,1263) = 42.89, *p* < 0.001, *η*^2^ = 0.004, *η*^2^*_*p*_* = 0.03. There was a significant interaction between creativity circumstances and profile (Figure 3), *F*(2,1263) = 87.67, *p* < 0.001, *η*^2^ = 0.02, *η*^2^*_*p*_* = 0.12. *Post hoc* comparisons revealed a significant increase of creativity for low-creativity participants, *t*(1263) = 11.62, *p*_*tukey*_ < 0.001, *d*_*RM*_ = 1.55, *M*_*diff*_ = 11.23, *SE* = 0.97. Medium participants also reported a significant increase of creativity, *t*(1263) = 5.55, *p*_*tukey*_ < 0.001, *d*_*RM*_ = 0.53, *M*_*diff*_ = 3.55, *SE* = 0.64. However, our results revealed the opposite effects for high creativity-participants (i.e., a significant decrease of creativity), *t*(1263) = −6.54, *p*_*tukey*_ < 0.001, *d*_*RM*_ = −0.825, *M*_*diff*_ = −5.46, *SE* = 0.89. Overall, these results were consistent with H2b. We observed also a weak but significant negative correlation between creativity differences and state boredom (*r* = −0.11, *p* < 0.001). Thus, H3b was not supported.

### Control Variables

To verify the effects of our control variables, we conducted two repeated measures ANCOVA, for professional and everyday creativity, with all our control variables as covariates. For Pro-C, the ANCOVA revealed no interaction between creativity differences and any of the covariates (for detailed results, see Supplementary Appendix D). Thus, it can be assumed that neither creative self-concept, personality factors, leisure time nor perceived difficulty of lockdown had an effect on the differences of Pro-C. For everyday creativity, the ANCOVA revealed a negative interaction between creativity differences and CSE, *β_*stand*_* = −0.12, *F*(1,1256) = 5.77, *p* < 0.05, and a positive interaction between creativity differences and CPI, *β_*stand*_* = 0.11, *F*(1,1256) = 5.22, *p* < 0.05. Results for everyday creativity are shown in Table 1. These results seem to indicate that the higher the CSE an individual possesses, the lower his or her gain in everyday creativity during lockdown. We see the opposite effect for CPI: the higher the CPI, the higher the gain in everyday creativity.

**TABLE 1:** Repeated measures ANCOVA for everyday creativity differences (within-subject effects).

	Sum of squares	df	Mean square	F	*p*
Creativity Differences	37.850	1	37.850	0.259	0.611
Creativity Differences * O	63.101	1	63.101	0.432	0.511
Creativity Differences * C	12.399	1	12.399	0.085	0.771
Creativity Differences * E	6.900	1	6.900	0.047	0.828
Creativity Differences * A	3.476	1	3.476	0.024	0.877
Creativity Differences * N	0.403	1	0.403	0.003	0.958
Creativity Differences * Leisure Time	311.128	1	311.128	2.132	0.144
Creativity Differences * Difficulty	9.222	1	9.222	0.063	0.802
Creativity Differences * CPI	762.019	1	762.019	5.222	0.022
Creativity Differences * CSE	842.186	1	842.186	5.771	0.016
Residual	183284.710	1256	145.927		

*Type 3 Sums of Squares. O, Openness; C, Conscientiousness; E, Extraversion; A, Agreeableness; N, Emotional Stability; CPI, Creative Personal Identity; CSE, Creative Self-Efficacy.*

## Discussion

Did lockdown lead to more creativity? This study aimed at answering this question and provides nuanced results to the scarce research on the potential benefits of the COVID-19 crisis. More precisely, we looked at professional and everyday creativity in France. Our results did not corroborate our hypotheses regarding Pro-C. In contrast, concerning everyday creativity, our results were consistent with our hypotheses.

Regarding Pro-C, our main hypothesis (H1a) was not supported by our results. Overall, contrary to our expectations, there was no increase in Pro-C during lockdown. Furthermore, no particular profile of Pro-C was more affected by the circumstances, as is evidenced by the fact that neither of the two identified profiles reported any changes in creativity during lockdown. These results appear to contradict recent research on the impact of uncertainty on creativity and innovation (Blauth et al., 2014; Beghetto, 2019), in the case of Pro-C. It may be the case that potential manifestations of Pro-C were impacted by an increase of anxiety during lockdown (e.g., Smith et al., 2020), which would be associated with a decrease in creativity (Byron and Khazanchi, 2011). In an exploratory fashion, we differentiated teleworkers from employees who continued to work in their usual worksite, expecting teleworkers to be more creative during lockdown, regarding their Pro-C. We observed the opposite: whereas teleworkers did not display increased Pro-C during lockdown, those who stayed at their usual worksite showed some increase, albeit characterized by a small-to-medium effect size (Cohen, 1988). These results contradict recent findings suggesting telework leads to higher creativity (Vega et al., 2015). For employees who stayed at their usual worksite and displayed increased creativity, it could be explained by the effect new constraints had on creativity (for a review, see Caniëls and Rietzschel, 2015). Indeed, whereas their usual worksites remained as they were before lockdown, work processes were most certainly disrupted and had to be rethought. These disruptions may have led to more creativity and innovation, in an effort to address them (Ford et al., 2008).

Regarding everyday creativity, in line with H1b, we did see an overall increase during lockdown, characterized by a small effect size. Furthermore, we differentiated three profiles of pre-lockdown everyday creativity, as a means to obtain a more nuanced perspective of the different impacts of lockdown on creativity. We found a very large effect size for low-creativity participants, and an intermediate effect size for medium-creativity participants. These results suggest that individuals with lower base-creativity prior to lockdown may have used this situation as an opportunity to be more creative in their personal lives, perhaps in reaction to the aforementioned negative experiences that ensued from lockdown measures in France. In contrast, we observed a large adverse effect for high-creativity participants. It seems that individuals already ahead in terms of everyday creativity prior to lockdown had more difficulty with the circumstances. This counter-intuitive result is corroborated by the negative interaction between everyday creativity differences and CSE, suggesting that individuals lower in CSE saw higher gains from the lockdown. It may be the case that the disruption of everyday life reduced the pre-existing “optimal” conditions for highly creative individuals. Additionally, we found an interesting significant negative correlation between the differences of everyday creativity and state boredom. Prior to analysis, we hypothesized that more boredom should lead to increased creativity, thus considering boredom as an antecedent of creativity, as has been conceptualized in the literature (Gasper and Middlewood, 2014; Mann and Cadman, 2014). However, our results show the opposite result and might warrant an alternative interpretation; individuals who displayed more creativity during lockdown were less bored by these circumstances. Encouraging people to pursue more creative activities might then be an effective way to reduce the burden of strict lockdown measures, in case of future lockdowns in France (Faranda and Alberti, 2020; Salje et al., 2020) or in other countries in general.

Although this study provides interesting contributions to the literature, it has several limitations. First, we used only self-report measures of creativity, which are satisfactory but not ideal (Ng and Feldman, 2012). In professional settings, it may be interesting to use supervisor-rated measures of creativity (Zhou and George, 2001), as they have proven to be more valid. Regarding everyday creativity, we opted for a shorter measure of creativity, as usual measures can be rather long and taxing on respondents (Galesic and Bosnjak, 2009). However, future studies are encouraged to take a more focused approach on individuals’ engagement in different creative activities and domains, using measures such as the recent Inventory of Creative Activities and Achievements (ICAA, Diedrich et al., 2018). This would certainly prove useful and provide more detailed knowledge on the impact of lockdown measures on the multiple domains of everyday creativity. A second limitation concerns the generalizability of our study. Whereas lockdown measures and policies might be similar across countries, their application and effectiveness remain dependent on the specificity of each country. Cross-cultural studies of the positives of lockdown measures are thus encouraged as well.

Overall, this study provides new and substantial insights into the consequences of lockdown measures. Lockdown may have inarguably negative consequences on the physical and mental health of many, but its positive impact exists as well. Rather than a burden, it might be useful to see these measures as opportunities to explore new horizons and engage in creative actions.

## Data Availability Statement

The raw data supporting the conclusions of this article will be made available by the authors, without undue reservation.

## Ethics Statement

Ethical review and approval was not required for the study on human participants in accordance with the local legislation and institutional requirements. The patients/participants provided their written informed consent to participate in this study.

## Author Contributions

MM, FV, NP, EB, NB, FG, and TL participated in the study design and methodology and in the revision process. MM and FV recruited the participants. MM performed the statistical analyses and wrote the first draft of the manuscript. All authors contributed to the article and approved the submitted version.

## Conflict of Interest

The authors declare that the research was conducted in the absence of any commercial or financial relationships that could be construed as a potential conflict of interest.

## Footnotes

1 According to this procedure, the scale’s items were translated into French by a first translator, then back into English by a second translator, to check whether the translation was correct or not.

## References

[B1] Akaike H. (1998). “Information theory and an extension of the maximum likelihood principle,” in *Selected Papers of Hirotugu Akaike*, eds Parzen E. Tanabe K. Kitagawa G. (New York, NY: Springer), 199–213. 10.1007/978-1-4612-1694-0_15

[B2] Akogul S. Erisoglu M. (2017). An approach for determining the number of clusters in a model-based cluster analysis. *Entropy* 19:452 10.3390/e19090452

[B3] Alfano V. Ercolano S. (2020). The efficacy of lockdown against COVID-19: a cross-country panel analysis. *Appl. Health Econ. Health Policy* 18 509–517. 10.1007/s40258-020-00596-3 32495067 PMC7268966

[B4] Alge B. J. Ballinger G. A. Tangirala S. Oakley J. L. (2006). Information privacy in organizations: empowering creative and extrarole performance. *J. Appl. Psychol.* 91:221. 10.1037/0021-9010.91.1.221 16435952

[B5] Amabile T. M. Hadley C. N. Kramer S. J. (2002). Creativity under the gun. *Harv. Bus. Rev.* 80 52–63. 12195920

[B6] Arieti S. (1976). *Creativity: The Magic Synthesis.* New York, NY: Basic Books Inc. Publishers.

[B7] Banerjee D. Rai M. (2020). Social isolation in Covid-19: the impact of loneliness. *Int. J. Soc. Psychiatry* 66 525–527. 10.1177/0020764020922269 32349580 PMC7405628

[B8] Banfield J. D. Raftery A. E. (1993). Model-based Gaussian and non-Gaussian clustering. *Biometrics* 49 803–821. 10.2307/2532201

[B9] Barabasz M. (1991). “Imaginative involvement in Antarctica: applications to life in space,” in *From Antarctica to Outer Space*, eds Harrison A. A. Clearwater Y. A. McKay C. P. (New York, NY: Springer), 209–215. 10.1007/978-1-4612-3012-0_19

[B10] Beghetto R. A. (2019). “Structured uncertainty: how creativity thrives under constraints and uncertainty,” in *Creativity Under Duress in Education?* Creativity Theory and Action in Education, Vol. 3 ed. Mullen C. (Cham: Springer), 27–40. 10.1007/978-3-319-90272-2_2

[B11] Beghetto R. A. Kaufman J. C. (2007). Toward a broader conception of creativity: a case for” mini-c” creativity. *Psychol. Aesthet. Creat. Arts* 1:73 10.1037/1931-3896.1.2.73

[B12] Besemer S. P. Treffinger D. J. (1981). Analysis of creative products: review and synthesis. *J. Creat. Behav.* 15 158–178. 10.1002/j.2162-6057.1981.tb00287.x

[B13] Biernacki C. Govaert G. (1997). Using the classification likelihood to choose the number of clusters. *Comput. Sci. Stat.* 29 451–457.

[B14] Blauth M. Mauer R. Brettel M. (2014). Fostering creativity in new product development through entrepreneurial decision making. *Creat. Innov. Manag.* 23 495–509. 10.1111/caim.12094

[B15] Brislin R. W. (1986). “The wording and translation of research instruments,” in *Field Methods in Cross-Cultural Research*, eds Lonner W. J. Berry J. W. (Beverly Hills, CA: Sage Publications, Inc), 137–164.

[B16] Brodeur A. Clark A. E. Fleche S. Powdthavee N. (2020). Assessing the impact of the coronavirus lockdown on unhappiness, loneliness, and boredom using Google Trends. *arXiv* [Preprint] arXiv:2004.12129,

[B17] Brooks S. K. Webster R. K. Smith L. E. Woodland L. Wessely S. Greenberg N. (2020). The psychological impact of quarantine and how to reduce it: rapid review of the evidence. *Lancet* 395 912–920. 10.1016/S0140-6736(20)30460-8 32112714 PMC7158942

[B18] Byron K. Khazanchi S. (2011). A meta-analytic investigation of the relationship of state and trait anxiety to performance on figural and verbal creative tasks. *Pers. Soc. Psychol. Bull.* 37 269–283. 10.1177/0146167210392788 21239599

[B19] Caniëls M. C. Rietzschel E. F. (2015). Organizing creativity: creativity and innovation under constraints. *Creat. Innov. Manag.* 24 184–196. 10.1111/caim.12123

[B20] Cavanaugh J. E. (1999). A large-sample model selection criterion based on Kullback’s symmetric divergence. *Stat. Probab. Lett.* 42 333–343. 10.1016/S0167-7152(98)00200-4

[B21] Chen S. Yao N. Qian M. (2018). The influence of uncertainty and intolerance of uncertainty on anxiety. *J. Behav. Ther. Exp. Psychiatry* 61 60–65. 10.1016/j.jbtep.2018.06.005 29909250

[B22] Cohen J. (1988). *Statistical Power Analysis for the Social Sciences.* Hillsdale, NJ: Lawrence Erlbaum Associates.

[B23] Cotter K. N. Christensen A. P. Silvia P. J. (2018). “Creativity’s role in everyday life,” in *Cambridge Handbook of Creativity*, 2nd Edn, eds Kaufman J. C. Sternberg R. J. (New York, NY: Cambridge University Press).

[B24] Counsell A. Furtado M. Iorio C. Anand L. Canzonieri A. Fine A. (2017). Intolerance of uncertainty, social anxiety, and generalized anxiety: differences by diagnosis and symptoms. *Psychiatry Res.* 252 63–69. 10.1016/j.psychres.2017.02.046 28254577

[B25] Cropley D. H. (2015). “Teaching engineers to think creatively: barriers and challenges in STEM disciplines,” in *The Routledge International Handbook of Research on Teaching Thinking*, eds Wegerif R. Li L. Kaufman J. C. (New York, NY: Routledge), 402–410.

[B26] Diedrich J. Jauk E. Silvia P. J. Gredlein J. M. Neubauer A. C. Benedek M. (2018). Assessment of real-life creativity: the inventory of creative activities and achievements (ICAA). *Psychol. Aesthet. Creat. Arts* 12:304 10.1037/aca0000137

[B27] Faranda D. Alberti T. (2020). Modelling the second wave of COVID-19 infections in France and Italy via a Stochastic SEIR model. *arXiv* [Preprint] ArXiv:2006.05081. 10.1063/5.0015943 33261336

[B28] Farmer R. Sundberg N. D. (1986). Boredom proneness–the development and correlates of a new scale. *J. Pers. Assess.* 50 4–17. 10.1207/s15327752jpa5001_2 3723312

[B29] Fletcher G. Griffiths M. (2020). Digital transformation during a lockdown. *Int. J. Inf. Manag.* 55:102185. 10.1016/j.ijinfomgt.2020.102185 32836642 PMC7333595

[B30] Ford C. M. (1996). A theory of individual creative action in multiple social domains. *Acad. Manag. Rev.* 21 1112–1142. 10.2307/259166

[B31] Ford C. M. Sharfman M. P. Dean J. W. (2008). Factors associated with creative strategic decisions. *Creat. Innov. Manag.* 17 171–185. 10.1111/j.1467-8691.2008.00486.x

[B32] Galesic M. Bosnjak M. (2009). Effects of questionnaire length on participation and indicators of response quality in a web survey. *Public Opin. Q.* 73 349–360. 10.1093/poq/nfp031

[B33] Gasper K. Middlewood B. L. (2014). Approaching novel thoughts: understanding why elation and boredom promote associative thought more than distress and relaxation. *J. Exp. Soc. Psychol.* 52 50–57. 10.1016/j.jesp.2013.12.007

[B34] Gaylin W. (1979). *Feelings: Our Vital Signs.* New York, NY: Harper and Row.

[B35] Gibson W. A. (1959). Three multivariate models: factor analysis, latent structure analysis, and latent profile analysis. *Psychometrika* 24 229–252. 10.1007/BF02289845

[B36] Harman H. H. (1976). *Modern Factor Analysis.* Chicago, IL: University of Chicago press.

[B37] Hill E. J. Hawkins A. J. Miller B. C. (1996). Work and family in the virtual office: perceived influences of mobile telework. *Fam. Relat.* 45 293–301. 10.2307/585501

[B38] Holt-Lunstad J. Smith T. B. Baker M. Harris T. Stephenson D. (2015). Loneliness and social isolation as risk factors for mortality: a meta-analytic review. *Perspect. Psychol. Sci.* 10 227–237. 10.1177/1745691614568352 25910392

[B39] Hunter J. A. Dyer K. J. Cribbie R. A. Eastwood J. D. (2016). Exploring the utility of the multidimensional state boredom scale. *Eur. J. Psychol. Assess.* 32:241 10.1027/1015-5759/a000251

[B40] Karwowski M. (2012). Did curiosity kill the cat? Relationship between trait curiosity, creative self-efficacy and creative personal identity. *Eur. J. Psychol.* 8 547–558. 10.5964/ejop.v8i4.513

[B41] Karwowski M. (2016). The dynamics of creative self-concept: changes and reciprocal relations between creative self-efficacy and creative personal identity. *Creat. Res. J.* 28 99–104. 10.1080/10400419.2016.1125254

[B42] Karwowski M. Barbot B. (2016). “Creative self-beliefs: their nature, development, and correlates,” in *Creativity and Reason in Cognitive Development*, eds Kaufman J. Baer J. (Cambridge: Cambridge University Press), 10.1017/CBO9781139941969.016

[B43] Karwowski M. Lebuda I. Wiśniewska E. (2018). Measuring creative self-efficacy and creative personal identity. *Int. J. Creat. Probl. Solving* 28 45–57.

[B44] Kaufman J. C. Beghetto R. A. (2009). Beyond big and little: the four C model of creativity. *Rev. Gen. Psychol.* 13 1–12. 10.1037/a0013688

[B45] Kaufman J. C. Beghetto R. A. (2013). In praise of clark kent: creative metacognition and the importance of teaching kids when (not) to be creative. *Roeper Rev.* 35 155–165. 10.1080/02783193.2013.799413

[B46] Koch P. (1994). *Solitude: A Philosophical Encounter.* Chicago, IL: Open Court Publishing.

[B47] Long C. R. Averill J. R. (2003). Solitude: an exploration of benefits of being alone. *J. Theory Soc. Behav.* 33 21–44. 10.1111/1468-5914.00204

[B48] Mann S. Cadman R. (2014). Does being bored make us more creative? *Creat. Res. J.* 26 165–173. 10.1080/10400419.2014.901073

[B49] Meinel M. Wagner T. F. Baccarella C. V. Voigt K.-I. (2019). Exploring the effects of creativity training on creative performance and creative self-efficacy: evidence from a longitudinal study. *J. Creat. Behav.* 53 546–558. 10.1002/jocb.234

[B50] Muthén B. O. (2001). “Latent variable mixture modeling,” in *New Developments and Techniques in Structural Equation Modeling*, eds G. A. Marcoulides and R. E. Schumacker (London: Psychology Press), 21–54.

[B51] Ng T. W. Feldman D. C. (2012). A comparison of self-ratings and non-self-report measures of employee creativity. *Hum. Relat.* 65 1021–1047. 10.1177/0018726712446015

[B52] Nitschke J. P. Forbes P. Ali N. Cutler J. Apps M. A. Lockwood P. (2020). Resilience during uncertainty. greater social connectedness during COVID-19 lockdown is associated with reduced distress and fatigue. *PsyArXiv* [Preprint] 10.31234/osf.io/9ehm7 PMC8247344 33099800

[B53] Perlman D. Peplau L. A. (1981). Toward a social psychology of loneliness. Personal Relationships, 3, 31–56.

[B54] Peters A. McEwen B. S. Friston K. (2017). Uncertainty and stress: why it causes diseases and how it is mastered by the brain. *Prog. Neurobiol.* 156 164–188. 10.1016/j.pneurobio.2017.05.004 28576664

[B55] Podsakoff P. M. Organ D. W. (1986). Self-reports in organizational research: problems and prospects. *J. Manag.* 12 531–544. 10.1177/014920638601200408

[B56] Pullano G. Valdano E. Scarpa N. Rubrichi S. Colizza V. (2020). Population mobility reductions during COVID-19 epidemic in France under lockdown. *medRxiv* [Preprint] 10.1101/2020.05.29.20097097 PMC7598368 33163951

[B57] Rosenberg J. Beymer P. Anderson D. van Lissa C. J. Schmidt J. (2019). tidyLPA: an R package to easily carry out latent profile analysis (LPA) using open-source or commercial software. *J. Open Source Softw.* 4:978 10.21105/joss.00978

[B58] Runco M. A. (1988). Creativity research: originality, utility, and integration. *Creat. Res. J.* 1, 1–7. 10.1080/10400418809534283

[B59] Runco M. A. Illies J. J. Eisenman R. (2005). Creativity, originality, and appropriateness: what do explicit instructions tell us about their relationships? *J. Creat. Behav.* 39 137–148. 10.1002/j.2162-6057.2005.tb01255.x

[B60] Runco M. A. Jaeger G. J. (2012). The standard definition of creativity. *Creat. Res. J.* 24 92–96. 10.1080/10400419.2012.650092

[B61] Salje H. Kiem C. T. Lefrancq N. Courtejoie N. Bosetti P. Paireau J. (2020). Estimating the burden of SARS-CoV-2 in France. *Science* 369 208–211. 10.1126/science.abc3517 32404476 PMC7223792

[B62] Schwarz G. (1978). Estimating the dimension of a model. *Ann. Stat.* 6 461–464. 10.1214/aos/1176344136

[B63] Simonton D. K. (2000). Creativity: cognitive, personal, developmental, and social aspects. *Am. Psychol.* 55 151–158. 10.1037//0003-066x.55.1.151 11392859

[B64] Smith L. E. Amlôt R. Lambert H. Oliver I. Robin C. Yardley L. (2020). Factors associated with self-reported anxiety, depression, and general health during the UK lockdown; a cross-sectional survey. *medRxiv* [preprint] 10.1101/2020.06.23.20137901 PMC7474581 32898760

[B65] Spores J. M. (1991). Developmental and behavioral correlates of loneliness among children and adolescents. *Diss. Abstr. Int.* 52:534B.

[B66] Storme M. Tavani J.-L. Myszkowski N. (2016). Psychometric properties of the French ten-item personality inventory (TIPI). *J. Individ. Differ.* 37 81–87. 10.1027/1614-0001/A000204

[B67] Tierney P. Farmer S. M. (2011). Creative self-efficacy development and creative performance over time. *J. Appl. Psychol.* 96:277. 10.1037/a0020952 20954756

[B68] Toohey P. (2011). *Boredom: A Lively History.* New Haven, CT: Yale University Press.

[B69] Vega R. P. Anderson A. J. Kaplan S. A. (2015). A within-person examination of the effects of telework. *J. Bus. Psychol.* 30 313–323. 10.1007/s10869-014-9359-4

[B70] Vodanovich S. J. (2003). Psychometric measures of boredom: a review of the literature. *J. Psychol.* 137 569–595. 10.1080/00223980309600636 14992349

[B71] Waples E. P. Friedrich T. L. (2011). Managing creative performance: important strategies for leaders of creative efforts. *Adv. Dev. Hum. Resour.* 13 366–385. 10.1177/1523422311424713

[B72] World Health Organization (2020). *Mental Health and Psychosocial Considerations During the COVID-19 Outbreak.* Available online at: https://apps.who.int/iris/handle/10665/331490 (accessed March 18, 2020).

[B73] Zhou J. George J. M. (2001). When job dissatisfaction leads to creativity: encouraging the expression of voice. *Acad. Manag. J.* 44 682–696. 10.5465/3069410 3069410

