# Supplementary material for: COVID-19: A Boon or a Bane for Creativity?
Source: Front Psychol. 2021 Jan 18;11:601150. doi: 10.3389/fpsyg.2020.601150 (PMC7848087; doi:10.3389/fpsyg.2020.601150)
Supplement: Supplementary file 1 [file Table_1.DOCX]

**Appendix A**

**Table A1**

*Everyday creativity scale in French and English*

|  | **Everyday creativity scale in French** | **English translation** |
| --- | --- | --- |
| **1.** | Je suggère de nouvelles façons pour atteindre mes buts ou mes objectifs. | I suggest new ways to achieve my goals or objectives. |
| **2.** | Je trouve des idées pratiques et nouvelles. | I come up with new and practical ideas. |
| **3.** | Je cherche de nouvelles manières de faire. | I search out new ways of doing things. |
| **4.** | Je suggère de nouvelles manières d'améliorer mon quotidien. | I suggest new ways to improve my everyday life. |
| **5.** | Je suis une bonne source d'idées créatives. | I am a good source of creative ideas. |
| **6.** | Je n'ai pas peur de prendre des risques. | I am not afraid to take risks. |
| **7.** | Je promeus et défends des idées aux autres. | I promote and champion ideas to others. |
| **8.** | Je fais preuve de créativité au quotidien quand j'en ai l'opportunité. | I exhibit creativity when given the opportunity to. |
| **9.** | J'ai souvent des idées nouvelles et innovantes. | I often have new and innovative ideas. |
| **10.** | Je trouve des solutions créatives aux problèmes. | I come up with creative solutions to problems. |
| **11.** | J'ai souvent une approche fraîche face aux problèmes. | I often have a fresh approach to problems. |
| **12.** | Je suggère de nouvelles façons de mener à bien ce que j'ai à faire. | I suggest new ways of carrying out what I have to do. |

**Appendix B**

**Table B1**

*Inter-items correlation matrix - TIPI*

|  | **E1** | | **E2R** | | **A1** | | **A2R** | | **C1** | | **C2R** | | **N1** | | **N2R** | | **O1** | | **O2R** | |
| --- | --- | --- | --- | --- | --- | --- | --- | --- | --- | --- | --- | --- | --- | --- | --- | --- | --- | --- | --- | --- |
| **E1** | — |  |  |  |  |  |  |  |  |  |  |  |  |  |  |  |  |  |  |  |
| **E2R** | **0.438** | ******* | — |  |  |  |  |  |  |  |  |  |  |  |  |  |  |  |  |  |
| **A1** | 0.007 |  | 0.013 |  | — |  |  |  |  |  |  |  |  |  |  |  |  |  |  |  |
| **A2R** | -0.346 | *** | -0.167 | *** | **0.132** | ******* | — |  |  |  |  |  |  |  |  |  |  |  |  |  |
| **C1** | 0.046 |  | -0.097 | *** | -0.121 | *** | -0.241 | *** | — |  |  |  |  |  |  |  |  |  |  |  |
| **C2R** | 0.051 |  | 0.039 |  | -0.218 | *** | -0.099 | *** | **0.279** | ******* | — |  |  |  |  |  |  |  |  |  |
| **N1** | -0.121 | *** | -0.139 | *** | 0.279 | *** | 0.055 |  | -0.020 |  | -0.170 | *** | — |  |  |  |  |  |  |  |
| **N2R** | 0.027 |  | 0.137 | *** | 0.232 | *** | 0.108 | *** | -0.186 | *** | -0.121 | *** | **0.408** | ******* | — |  |  |  |  |  |
| **O1** | 0.344 | *** | 0.115 | *** | -0.008 |  | -0.225 | *** | 0.118 | *** | 0.099 | *** | -0.161 | *** | -0.049 |  | — |  |  |  |
| **O2R** | 0.186 | *** | 0.200 | *** | -0.060 | * | -0.117 | *** | 0.009 |  | 0.122 | *** | -0.105 | *** | 0.046 |  | **0.239** | ******* | — |  |
| *Note 1.* * p < .05, ** p < .01, *** p < .001*.* | | | | | | | | | | | | | | | | | | | | |
| *Note 2.* Relevant inter-items correlations are shown in bold.  *Note 3*. E : Extraversion, A : Agreeableness, C : Conscientiousness, N : Emotional Stability, O : Openness. | | | | | | | | | | | | | | | | | | | | |

**Appendix C**

**Table C1**

*Latent profile analysis – Number of profiles comparison for professional creativity*

|  | | | | | |
| --- | --- | --- | --- | --- | --- |
| **Number of profiles** | **AIC** | **AWE** | **BIC** | **CLC** | **KIC** |
| **1** | 1802.21 | 1828.02 | 1811.12 | 1800.21 | 1807.21 |
| **2** | 1778.79 | 1833.22 | 1796.59 | 1771.97 | 1785.79 |
| **3** | 1776.00 | 1858.21 | 1802.71 | 1765.22 | 1785.00 |
| **4** | 1772.41 | 1882.24 | 1808.03 | 1757.82 | 1785.41 |
| **5** | 1770.91 | 1908.51 | 1815.43 | 1752.36 | 1783.91 |
| **6** | 1774.68 | 1940.27 | 1828.11 | 1751.94 | 1789.68 |
| **7** | 1778.60 | 1972.04 | 1840.93 | 1751.81 | 1795.60 |

**Table C2**

*Latent profile analysis – Number of profiles comparison for everyday creativity*

|  | | | | | |
| --- | --- | --- | --- | --- | --- |
| **Number of profiles** | **AIC** | **AWE** | **BIC** | **CLC** | **KIC** |
| **1** | 3604.27 | 3662.85 | 3614.56 | 3602.27 | 3609.27 |
| **2** | 3570.82 | 3630.94 | 3591.40 | 3563.87 | 3577.82 |
| **3** | 3558.84 | 3649.37 | 3589.71 | 3548.06 | 3567.84 |
| **4** | 3562.44 | 3683.68 | 3603.60 | 3547.53 | 3573.44 |
| **5** | 3566.33 | 3718.22 | 3617.79 | 3547.37 | 3579.33 |
| **6** | 3556.30 | 3738.51 | 3618.05 | 3533.59 | 3571.30 |
| **7** | 3559.93 | 3772.83 | 3631.97 | 3533.12 | 3576.93 |

**Appendix D**

**Table D1**

*Repeated measures ANCOVA for professional creativity differences (within-subject effects)*

|  | | | | | |
| --- | --- | --- | --- | --- | --- |
|  | **Sum of Squares** | **df** | **Mean Square** | **F** | **p** |
| Creativity Differences | 21.927 | 1 | 21.927 | 0.124 | 0.725 |
| Creativity Differences ✻ O | 1.121 | 1 | 1.121 | 0.006 | 0.937 |
| Creativity Differences ✻ C | 453.274 | 1 | 453.274 | 2.569 | 0.109 |
| Creativity Differences ✻ E | 19.517 | 1 | 19.517 | 0.111 | 0.740 |
| Creativity Differences ✻ A | 74.653 | 1 | 74.653 | 0.423 | 0.516 |
| Creativity Differences ✻ N | 22.186 | 1 | 22.186 | 0.126 | 0.723 |
| Creativity Differences ✻ Leisure Time | 2.873 | 1 | 2.873 | 0.016 | 0.899 |
| Creativity Differences ✻ Difficulty | 508.434 | 1 | 508.434 | 2.881 | 0.090 |
| Creativity Differences ✻ CPI | 24.166 | 1 | 24.166 | 0.137 | 0.711 |
| Creativity Differences ✻ CSE | 360.541 | 1 | 360.541 | 2.043 | 0.153 |
| Residual | 113987.098 | 646 | 176.451 |  |  |
| *Note 1.* Type 3 Sums of Squares*.*  *Note 2*. O : Openness, C : Conscientiousness, E : Extraversion, A : Agreeableness, N : Emotional Stability, CPI : Creative Personal Identity, CSE : Creative Self-Efficacy. | | | | | |
|  | | | | | |
